# Preoperative anxiety differentiates post‐surgical opioid use in patients undergoing anterior cruciate ligament reconstruction

**DOI:** 10.1002/jeo2.70352

**Published:** 2025-07-13

**Authors:** Brittany L. Nelson, Shayla M. Warren, Thea Xeroegeanes, Taylor M. Zuleger, Ajay Premkumar, Gregory D. Myer, Susmita Kashikar‐Zuck, Jed A. Diekfuss

**Affiliations:** ^1^ Emory Sports Performance And Research Center (SPARC) Flowery Branch Georgia USA; ^2^ Emory Sports Medicine Center Atlanta Georgia USA; ^3^ Department of Orthopaedics Emory University School of Medicine Atlanta Georgia USA; ^4^ Undergraduate Pre‐Nursing Studies University of Arkansas Fayetteville Arkansas USA; ^5^ Department of Veterans Affairs Atlanta VA Medical Center Decatur Georgia USA; ^6^ Youth Physical Development Centre Cardiff Metropolitan University Wales UK; ^7^ Wallace H. Coulter Department of Biomedical Engineering Georgia Institute of Technology & Emory University Atlanta Georgia USA; ^8^ Department of Pediatrics University of Cincinnati College of Medicine Cincinnati Ohio USA; ^9^ Division of Behavioral Medicine, Cincinnati College of Medicine Cincinnati Children's Hospital Medical Center Cincinnati Ohio USA

**Keywords:** opioid use, patient anxiety, post‐ACLR recovery, substance use disorder

## Abstract

**Purpose:**

Opioids prescribed to reduce pain and aid in recovery following anterior cruciate ligament reconstruction (ACLR) may pose a risk of future substance use disorder. The purpose of this study was to determine if pre‐operative anxiety and depression differentiated opioid intake following ACLR. The contribution of sex, age, and graft type to post‐operative opioid usage was also explored.

**Methods:**

Data from 237 participants (*M* = 30.75 ± 13.29 years; 57% females; 76% all‐soft tissue quadriceps tendon autograft) were analysed. Four‐item Anxiety and Depression Patient Reported Outcomes Measurement Information System (PROMIS) scales were administered on the day of surgery, and opioid intake was assessed post‐operatively. Patients were classified into 'anxious' or 'depressed' groups based on their PROMIS scores.

**Results:**

Patients took an average of one opioid pill daily. Females with pre‐operative anxiety reported significantly greater post‐operative opioid intake (*M* = 4.40 ± 3.98) than females with no anxiety (*M* = 2.90 ± 3.97) (*W* = 2199, *p* = 0.03; *d* = 0.36). No other significant effects were found (*p* > 0.05).

**Conclusion:**

Anxiety, but not depression, was a risk factor for elevated opioid use in females undergoing ACLR. Interestingly, opioid intake between males and females, as well as by age and graft type, were comparable overall, indicating the unique influence of psychological rather than biological or demographic factors on opioid use following ACLR. Clinicians should consider biopsychosocial assessments to support preoperative opioid counselling, particularly in females with anxiety undergoing ACLR.

**Level of Evidence:**

Level IV.

**Abbreviations**
ACL: anterior cruciate ligament
ACLR: anterior cruciate ligament reconstruction
ANOVA: analysis of variance
BMI: body mass index
CBT: cognitive behavioral therapy
CI: confidence interval
NSAIDs: non‐steroidal anti‐inflammatory drugs
OUD: opioid use disorder
PRO: patient‐reported outcome
PROMIS: Patient Reported Outcomes Measurement Information System
RCT: randomised controlled trial
SD: standard deviation
SEM: standard error of the mean
W: Mann–Whitney *U* test statistic

## INTRODUCTION

The anterior cruciate ligament (ACL), critical for knee stability, is frequently injured, with an annual incidence rate of approximately 29.6 per 100,000 people [16, 26]. ACL reconstruction (ACLR) postoperative knee pain is common, necessitating effective pain management to improve recovery and reduce complications [17, 27, 33, 35]. To enhance patient outcomes following ACLR, effective pain management is vital [2]. Currently, over 85% of practitioners prescribe opioids post‐ACLR to alleviate pain effectively [9, 34]. However, widespread opioid use carries a significant risk of opioid use disorder and overdose [8, 39].

Opioid consumption has numerous biopsychosocial (e.g., anxiety, gender and fear) and clinical (e.g., type of surgery and patient health status) risk factors. Although risk factors for OUD have been studied in patients undergoing total knee arthroplasty [3, 18, 21] and total hip arthroplasty [21], less is known about the risk factors for OUD following ACLR [6, 36]. There is a significant gap in the literature regarding patient risk factors of OUD for patients with ACLR [6, 36]. Given these knowledge gaps, it is important to consider how psychological factors such as anxiety and depression, particularly as they differ by sex, may contribute to variability in opioid use following ACLR.

Males and females have vast differences in the prevalence, severity and symptoms of anxiety and depression, with stronger symptoms and frequency among women compared to men [10, 32, 40]. Anxiety can exacerbate pain perception and increase the demand for pain medication [4, 22]. These studies, therefore, suggest that anxious females are more likely to use more opioids following surgery compared to males.

This study proposes to evaluate the potential of psychological risk factors on opioid use, particularly as they vary by sex. The novelty of this study is prospectively examining the influence of anxiety and depression (pre‐surgery) on post‐operative opioid intake in patients with ACLR, with a particular focus on how these potential risk factors differ by sex. Based on these objectives, the following hypotheses were: (a) female patients with pre‐operative anxiety and depression would report greater opioid use following ACLR compared to female patients without pre‐operative anxiety and depression and (b) male patients would report similar opioid use regardless of pre‐operative anxiety and depression.

## METHODS

### Participants

This study enroled 237 patients undergoing ACLR. Ten individuals were removed from analyses due to incomplete data, resulting in a final sample size of 227.

Patients were prescribed 15 Oxycodone opioid tablets (5 mg each) to manage post‐operative pain. The instructions on the bottle indicated that patients could take one tablet every 6 hours as needed. They were instructed by the surgeon at the time of care and by the post‐operative nurse to take opioids for post‐operative pain only if the pain was severe (≥7 on a 0–10 scale). Patients were given examples of when to take the opioids (e.g., if the pain was so severe that they could not eat their favourite food, sleep, or hang out with their favourite person). Side effects (e.g., constipation and nausea) and alternative pain management techniques (e.g., distraction) were also discussed in‐depth with each patient. Each patient was also placed on a scheduled dose of 1000 mg of Acetaminophen every 8 h and 500 mg of Naproxen every 12 h.

### Materials

The National Institutes of Health Patient Reported Outcomes Measurement Information System (PROMIS) anxiety and depression scales were used to evaluate patient anxiety and depression within the past 7 days [1]. PROMIS employs psychometric methods to develop industry‐standard patient‐reported outcome measures that are valid, reliable, and capable of distinguishing between individuals who score high versus low on specific traits [7]. The four‐item anxiety and depression short‐form scales were administered pre‐operatively on the day of surgery and have strong psychometric properties to measure anxiety and depression across patient populations [13, 15]. Scale responses were converted to standardised *t*‐scores based on normative data [23]. Patients with mild to severe anxiety and depression were grouped into overall 'anxious' or 'depressed' groups according to PROMIS cut‐points, which are based on normative data [23]. Patients who did not report mild to severe anxiety or depression were grouped into 'not anxious' or 'not depressed' groups, respectively.

Patients self‐reported how many opioid pills they took following surgery via a questionnaire asking, 'How many pills have you taken?' and 'How many pills do you have left?' (completed 4.32 ± 4.84 days post‐operatively).

### Statistical analyses

Analyses were performed using RStudio [37]. Descriptive statistics were calculated, including means, standard deviations, and proportions. The Shapiro–Wilk test was conducted for each group to assess the normality assumption for independent two‐sample *t*‐tests. The Mann–Whitney *U* test was used for all group comparisons because the data did not pass the Shapiro‐Wilk test for normality. Separate analyses were performed to compare opioid intake for those who were anxious and not anxious, as well as depressed and not depressed, for males and females separately. A Pearson's product‐moment correlation was used to assess relationships by opioid intake, and one‐way analysis of variance (ANOVA) examined differences in opioid use by graft type. The average number of pills taken per day, as reported at the time of the survey, was also noted.

## RESULTS

Participant demographic characteristics and psychological profiles are summarised in Tables 1 and 2. Table 1 provides an overview of the participants' age, sex distribution, and graft types. Table 2 details the distribution of anxiety and depression status for males and females.

**Table 1:** Patient demographics.

Demographic		
Age (years)		*M* = 30.75 ± 13.29
		Range: 12–63
Sex	Male	43% (*n* = 102)
	Female	57% (*n* = 135)
Graft type	Bone‐patellar tendon‐bone autograft	8% (*n* = 19)
	Tibialis anterior allograft	14% (*n* = 35)
	All‐soft tissue quadriceps tendon autograft	76% (*n* = 182)

**Table 2:** Distribution of participants based on anxiety and depression status.

Males	
Anxious	41.58% (*n* = 42)
Not anxious	58.42% (*n* = 59)
Depressed	7.84% (*n* = 7)
Not depressed	92.16% (*n* = 94)
Females	
Anxious	47.62% (*n* = 60)
Not anxious	52.38% (*n* = 66)
Depressed	21.43% (*n* = 27)
Not depressed	78.57% (*n* = 99)

### Anxiety

**Figure 1:**
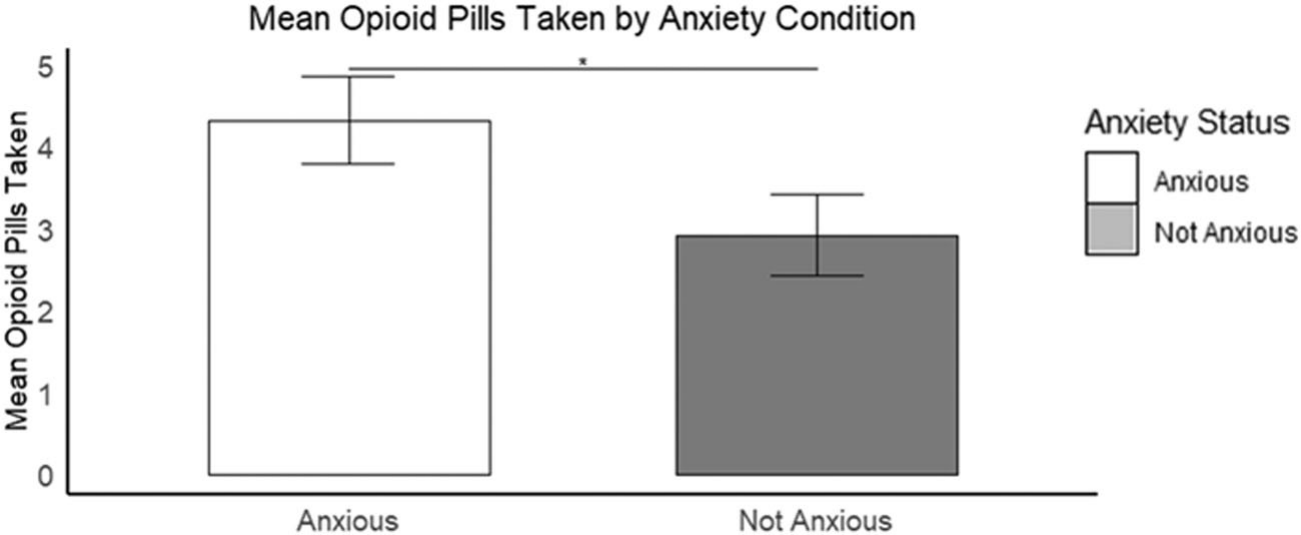
Bar plot with mean number of opioid pills taken among females by anxiety condition. Bars represent the average pill intake, with error bars indicating the standard error. *Indicates a statistically significant difference (*p* < 0.05).

Females with pre‐operative anxiety reported significantly greater post‐operative opioid intake (*M* = 4.40 ± 3.98) compared to females with no anxiety (*M* = 2.90 ± 3.97) (Mann–Whitney *U* test, *W* = 2199, *p* = 0.03; *d* = 0.36) (Figure 1). Males with pre‐operative anxiety did not report significantly greater post‐operative opioid intake (*M* = 3.06 ± 4.20) compared to males with no anxiety (*M* = 2.97 ± 3.48) (Mann–Whitney *U* test, *W* = 1108.5, *p* = 0.56; *d* = 0.03) (Figure 1).

### Depression

Females with pre‐operative depression did not report significantly greater post‐operative opioid intake (*M* = 3.87 ± 3.75) than females with no depression (*M* = 3.51 ± 4.07) (Mann–Whitney *U* test, *W* = 1335, *p* = 0.47; *d* = 0.36). Males with pre‐operative depression also did not report significantly greater post‐operative opioid intake (*M* = 4.29 ± 5.47) than males with no depression (*M* = 2.91 ± 3.64) (Mann‐Whitney U test, *W* = 349, *p* = 0.71; *d* = 0.09).

### Demographic risk factors

There was no statistically significant relationship between age and the number of pills taken (*r* = 0.08, *p* = 0.25). There was also no statistically significant difference in opioid intake between males (*M* = 3.01 ± 3.78) and females (*M* = 3.59 ± 3.99) (Mann–Whitney *U* test, *W* = 6486.5, *p* = 0.23; *d* = 0.15).

### Graft type

Means and standard deviations for post‐operative opioid intake per graft type were as follows: bone‐patellar tendon‐bone autograft (*M* = 3.66 ± 3.43), tibialis anterior allograft (*M* = 3.27 ± 3.89), and all‐soft tissue quadriceps tendon autograft (*M* = 3.24 ± 3.93). ANOVA revealed no significant differences in opioid intake between graft types (*F*(2, 222) = 0.10, *p* = 0.90, *η* ^2^ < 0.01).

### Opioid intake by day

**Figure 2:**
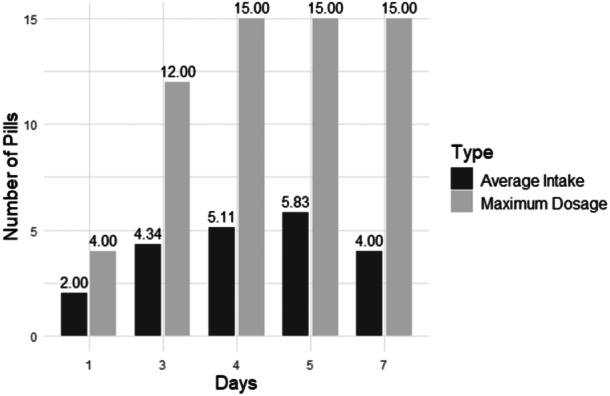
Average opioid pill intake after removing participants who took no opioids at the time of survey compared to maximum possible dosage.

Table 3 shows the average opioid intake by postoperative day. Overall, participants took an average of one pill per day at the time of the survey. When excluding participants who took no opioids following surgery, the average increased to two pills per day (see Figure 2).

**Table 3:** Mean, standard deviation, and size of the sample surveyed for opioid intake, based on the number of days after surgery participants were surveyed.

Day	*M*	SD	*N*
1	1	1.41	2
3	2.98	3.91	64
4	3.59	3.88	134
5	2.19	4.34	16
7	2	2.83	2
27	0	NA	1
43	0	NA	1
57	1	NA	1
NA	4.86	2.67	13

Abbreviations: NA, not assessed; SD, standard deviation.

## DISCUSSION

The most important finding of this study was that female patients with pre‐operative anxiety had significantly higher opioid intake following ACLR than females without anxiety. Other factors, such as depression, age, and graft type, did not significantly impact opioid use. These results demonstrate the importance of identifying and addressing psychological risk factors, particularly anxiety, to mitigate the potential for opioid misuse and addiction.

Consistent with the hypothesis, anxiety significantly impacted opioid intake in females following ACLR. This work parallels previous findings from total knee and hip arthroplasty, showing an association between anxiety and opioid intake [5, 14, 28]. Techniques designed to address anxiety, including mindfulness [11], relaxation exercises [20, 30] or cognitive‐behavioral therapy [20] may help mitigate opioid reliance in at‐risk patients. The results from this study underscore the importance of incorporating psychological screening into preoperative evaluations—particularly for female patients—as a potential strategy for identifying individuals who may require additional support during recovery. There is a need for additional research to support more data‐driven, surgery‐specific recommendations for opioid prescriptions that minimise post‐operative consumption while maintaining patient pain management.

Given that opioid dependence can develop after just five days of use [38], limiting opioid exposure is critical. While we did not systematically manipulate alternative pain treatments, these findings, in addition to prior research, indicate that a combination of scheduled non‐opioid pain relievers and patient education may effectively reduce opioid intake without compromising pain control [12, 19]. Orthopedic physician prescribing practices currently range from 15 to 60 opioid tablets following ACLR [29]. However, the data indicates that many patients take fewer opioids than prescribed, with an average intake of just one pill per day. This aligns with previous recommendations that 15 oxycodone 5‐mg tablets may be sufficient for post‐ACLR pain management [29], though further reductions could be achieved when paired with additional pain control methods such as nerve blocks [41]. The patients in this study received a preoperative adductor canal block, which likely contributed to the low opioid consumption within the first 12–24 h after surgery. Additionally, all patients followed a scheduled regimen of 1000 mg of acetaminophen every 8 h and 500 mg of naproxen every 12 h, further reducing the need for opioids.

Despite this study's contributions, there are several limitations. First, all patient data were collected from a single hospital, limiting generalisability. Future research should include data from multiple hospitals or regions to improve the applicability of results across diverse populations. Future longitudinal research is needed to examine temporal associations between these risk factors and opioid intake to understand causality better. The dataset also included very few patients with severe anxiety and no patients with severe depression, further limiting the generalisability of the findings. Future studies should also include a more balanced distribution of graft types (e.g., more bone‐patellar‐tendon bone and hamstring autografts) to assess their relative influence on opioid intake. Anxiety was measured pre‐operatively on the day of surgery, likely capturing the acute stress response or emotional response to the situation (state anxiety) as related to the surgery rather than stable personality characteristics (trait anxiety). A patient's state anxiety may impact pain perception and thus influence opioid intake [24, 25]. Future studies should consider measuring anxiety at multiple time points, including days other than the surgical procedure, to differentiate between state and trait anxiety. Finally, while depression did not significantly impact opioid intake in this study, future research should evaluate its potential effects over the prolonged rehabilitation period, particularly given the likelihood that patients experience depressive symptoms as a result of activity restrictions post‐surgery [31].

This study reveals a significant sex‐specific difference between pre‐operative anxiety and opioid consumption following ACLR, with anxious females taking significantly more opioids after surgery than non‐anxious females. These findings raise intriguing questions about whether managing pre‐operative anxiety in females could reduce post‐operative opioid intake and what other factors might influence opioid use after ACLR. Further research is needed to test targeted interventions that address pre‐operative anxiety to minimise opioid use and improve patient well‐being.

## AUTHOR CONTRIBUTIONS

All authors contributed to the study conception and design. Brittany Nelson wrote the first draft of the manuscript, and all authors commented on versions of the manuscript. Shayla M. Warren, Thea Xeroegeanes, Taylor M. Zuleger, Gregory D. Myer, and Jed A. Diekfuss performed material preparation, data collection, and analysis. Brittany L. Nelson led the statistical analysis. All authors read and approved the final manuscript.

## CONFLICT OF INTEREST STATEMENT

Gregory D. Myer consults with commercial entities to support commercialization strategies but has no direct financial interest in commercialization of the products. Dr. Myer's institution receives past, current and ongoing grant funding from National Institutes of Health/National Institute of Arthritis and Musculoskeletal and Skin Diseases grants (grants U01AR067997, R01AR070474, R01AR055563, R01AR076153, R01AR077248, and R61AT012421), the Department of Defense (W81XWH22C0062), Department of Veteran's Affairs (CReATE Motion Center), and the Arthritis Foundation Osteoarthritis Clinical Trial Network. Dr. Myer has received industry‐sponsored research funding to his institutions related to injury prevention and sport performance and has current ongoing funding from Arthrex Inc. to evaluate ACL surgical treatment optimization strategies. Dr. Myer receives author royalties from Human Kinetics and Wolters Kluwer. Dr. Myer is an inventor of biofeedback technologies (patent US11350854B2, Augmented and Virtual Reality for Sport Performance and Injury Prevention Application, approved 6/7/2022, software copyrighted) designed to enhance rehabilitation and prevent injuries, which has received licensing royalties. Dr. Diekfuss is also eligible to receive inventor‐related royalties from biofeedback technologies. Dr. Diekfuss also receives author royalties from Kendall Hunt Publishing Company. Dr. Kashikar‐Zuck receives funding from the National Institutes of Health/National Institute of Arthritis and Musculoskeletal and Skin Diseases grant P30 AR076316.

## ETHICS STATEMENT

This study was completed as a retrospective chart review and was approved by the institutional review board at Emory University (STUDY00008338). All authors consent to the publication of the manuscript.

## Data Availability

Data is available upon reasonable request.
